# A Perspective on Consumers 3.0: They Are Not Better Decision-Makers than Previous Generations

**DOI:** 10.3389/fpsyg.2016.00848

**Published:** 2016-06-03

**Authors:** Petr Houdek

**Affiliations:** ^1^Faculty of Business Administration, University of EconomicsPrague, Czech Republic; ^2^Faculty of Social and Economic Studies, Jan Evangelista Purkyně University, Ústí nad LabemCzech Republic; ^3^Center for Behavioral Experiments, PragueCzech Republic

**Keywords:** anchoring, decision-making biases, cognitive frames, consumer 3.0, limited attention, local thinking

## Abstract

This perspective article builds upon the theory of local thinking in interpretation and prediction of consumer behavior in a contemporary world of information overload. It is shown that even informed and socially and environmentally responsible consumers (consumers 3.0) exhibit selective recall, limited attention, and bounded search in the perception and interpretation of price and quality of purchases. Their decisions fall into local cognitive frames, which specifically focus attention only on a narrow structure and content of the choice. The cognitive frames can be established by recent or regular purchases, but also extreme or primary purchase experiences. The article includes a short conceptual review of car, food, clothing, insurance, drugs, paintings, and other product purchases showing that the local cognitive frames often lead to bad bargains across various sectors. The article presents several suggestions for future research.

## Introduction

The aim of this perspective article is to show that even in age of an informed and socially and environmentally responsible consumer (consumer 3.0), his/her style of decision-making has not changed much. People still tend to overrate the importance of the information that is vivid, emotional, recent, or frequent and therefore easily appears in their minds, as Tversky and Kahneman (1974, p. 1127) famously wrote: “It is a common experience that the subjective probability of traffic accidents rises temporarily when one sees a car overturned by the side of the road.” The immediate experience creates a specific cognitive frame under which people interpret further information. In an insurance choice the consumers thus prefer insurance for a high probability, low consequence risks, and downplay insurance for a low probability, high consequence risks (Browne et al., 2015), or that insurance take-up spikes the year after a catastrophe and then steadily declines as salience of the event fades off (Gallagher, 2014). Consumers are framed by immediate weather to buy goods with advantageous attributes in that particular weather, underestimating different future circumstances or changes in their preferences, followed by high returns rates (Conlin et al., 2007; Busse et al., 2015). Examples of other cognitive frameworks are abundant.

This article elaborates the concept of cognitive frames in consumer research to offer a simple unifying framework of the forces influencing consumer decisions in contemporary complex consumer environment (see also Olshavsky and Granbois, 2002; Uncles et al., 2002; Foxall, 2010). I utilize a behavioral theory about consumers’ local thinking that has been conceptualized in a series of articles by Gennaioli and Shleifer (2010) and Bordalo et al. (2012; 2013; 2015) and is itself inspired by a model of selective recall (Kahneman and Miller, 1986). This approach offers a fruitful conceptualization of consumer decision-making across the retail sector.

The article follows by showing that consumers do not consider all relevant information when shopping. Their behavior is better described by selective recall, limited attention, and bounded search. In the third section a theory of consumer local thinking is introduced. When demonstrating its relevance, I particularly refer to contemporary studies about consumer behavior in market settings to avoid criticism of laboratory and/or survey research. Although laboratory-based studies have proved useful in measuring many aspects of shopping cognition and behavior, they may suffer from problems of low external validity and non-replicability, their participants do not necessarily represent broader populations, and it is generally difficult to capture highly dynamic and incentivized aspects of markets in a lab (Hertwig and Ortmann, 2001; Henrich et al., 2010; Open Science Collaboration, 2015).

## Limited Search and Attention

The cognitive frames approach of consumer decision-making is interlinked with the findings that consumers’ willingness (or capability) to seek out new information, their attention and memory are not limitless (DellaVigna, 2009; Iyengar and Kamenica, 2010; Houdek and Koblovský, 2015) and consumers may not realize these aspects of their bounded rationality (but there is a great heterogeneity in consumers’ shopping sophistication). Moreover, the natural reaction of firms to bounded rationality of some consumers is to make their price policies more complex and less transparent to further hamper consumers’ ability to compare products (Hortaçsu and Syverson, 2004; Carlin, 2009). In many market conditions there is no reason to expect that competition among firms will push to greater price transparency, e.g., if median consumers focus only on a base price and ignore shrouded add-ons, then firms with transparent pricing lose to non-transparent firms (Gabaix and Laibson, 2006).

Consumers focus only on a small fraction of products. An average consumer is deciding between two to five cars while 160 brands of them exist; when there are flooding coffee options consumers are making a decision only between three and four alternatives – essentially regardless of the range of possible substitutes, a consumer takes into account always only a few units of alternatives (Hauser and Wernerfelt, 1990). The advent of internet search engines and comparative sites dramatically increased opportunities to choose the best deals by allowing consumers to engage in low-cost price comparisons (Brown and Goolsbee, 2002; Zettelmeyer et al., 2006), nevertheless the prices on the internet are far from converging to the law of one price.

The amount of time spent on shopping (including browsing and buying) has not changed at all in more than 30 years (Ott, 2011). Consumers are still affected by the most salient sales (Grubb, 2015). Additionally, “advances in search technology are accompanied by investments by firms in obfuscation” (Ellison and Ellison, 2009, p. 427). Ellison and Ellison show that charging a low price for a low-quality product on a price comparison website actually increases retailer’s sales of medium- and high-quality products on the retailer’s own website. Consumers are influenced by the salient (top) positions of low-quality product on comparison website, they move to retailer own website, where they are persuaded to buy a higher-quality higher-price product instead (see also Chioveanu and Zhou, 2013 and Spiegler, 2014).

Moreover, complex pricing policies and (perceived) quality differences of many product categories do not always allow direct comparison (Greenleaf et al., 2016). Consumers must rely on limited search and are thus heavily influenced by various levels of information disclosure (Brown et al., 2010) and/or advertising, which exacerbate their confusion. Consumers’ take-up of credit card offers responds much less to the interest rate on credit cards than to a photo of an attractive woman in a mailing marketing campaign (Bertrand et al., 2010). Consumers are willing to pay high premium for branded product, which is homogeneous to non-branded alternatives. In their case study of headache remedies Bronnenberg et al. (2015) found that more informed consumers (such as physicians and pharmacists) choose national brands over store brands only 9% of the time, compared to 26% of the time for the average consumer.

Consumers pay attention largely to digits that are on the left in a string of numbers. The left-digit bias creates a salient break-point, which is why items priced at 0.99 or 3.99 dollars are considered much better deals than items priced at 1.00 or 4.00 dollars. The bias is not governing only trivial purchases. At American used-car market Lacetera et al. (2012) found that vehicles with odometer values between 79,900 and 79,999 miles were sold on average for approximately 200 dollars more than cars with odometer values between 80,000 and 80,100 miles, but for only 10 dollars less than cars with odometer values between 79,800 and 79,899. Such focal points could be found in a number of consumer choices. Scott and Yelowitz (2010) detected that prospective grooms are willing to pay premiums upward of 18% for an engagement ring’s diamond that is one-half carat rather than slightly less than a half carat and between 5 and 10% for a one-carat rather than a slightly less than one-carat stone.

When price promotions occur, many consumers fail to switch to purchasing a package that has the lowest unit price, which suggests a lack of price awareness. In their study on quantity surcharges Clerides and Courty (2015) showed a decrease of only 27% in the sales of disadvantageous large laundry detergents relative to the sales the week preceding the surcharge. In the utilities sector, households rarely search for alternative retailers, and when they do search, the current retailer has a significant brand advantage (Hortaçsu et al., 2015). Nevertheless, real-time feedback on quantity of electricity consumed via an in-home display could have a substantial impact on price elasticity of households (Jessoe and Rapson, 2014). The advent of technology using big data about consumption could lead to higher price and quality transparency. It could be expected that consumers’ sensitivity to price and quality features of products will grow.

## Consumers’ Local Cognitive Frames

Cognitive frames create simplified mental models of choices. They constrain how consumers respond to stimuli by selectively organizing information from the consumers’ environment and by focusing attention only on a narrow structure and content of the perceived attributes of a choice. They thus create specific expectations about a product’s price or consumption quality (Gennaioli and Shleifer, 2010; Bordalo et al., 2013, 2015). For example, a classic study by Hoyer (1984) found that while choosing a laundry detergent, consumers examine on average only 1.4 packages and only 11% of them look at more than two. Hoyer concluded that 91% of the consumers were governed by a simple, one-statement reason for their choice (e.g., “I like it” tactics).

The expectations about prominent attributes of a product, or consideration sets (Roberts and Lattin, 1997; Houdek, 2016), are at the top of a consumer’s mind and they lead to a particular interpretation of a choice at hand and affect the alternatives consumers consider (Tversky et al., 1988). Other aspects of the choice are reflected or recalled only partially or not at all. This neglect makes consumers leave out better products from their consideration sets.

The expectations (cognitive frames) can be created by (i) deeply remembered psychological states – strong personal or primary experiences could create a general tendency for evaluating all following products and create a long lasting consumption pattern (see Strong and Primary Memories, Attribution Error). They are further formed by (ii) recalls from immediate past or from frequent consumption (see Immediate and Frequent Consumption, Anchoring Bias); the more often or saliently consumers were experiencing some attribute of choice, the stronger cognitive frame of this choice they then apply.

The expectations could originate from (iii) transient mood and affects (see Situational Influences, Emotion, and Mood, Affect Bias) or just in other words (iv) they can be created by momentarily salient external circumstances; typically, from information supplied by marketing devices or projections of current tastes influenced by immediate context to the future (see Situational Influences and Projection Error). Moreover, an attribute of choice options that is different from the expectations is most salient and the consumer overrates it in their decision-making.

### Strong and Primary Memories, Attribution Error

Expectations could be derived from primary and/or extreme experiences that consumers more easily recall from memory. The most apparent display of this influence is the loyalty of a consumer to a brand, which originates along with feelings of a satisfactory purchase (Chaudhuri and Holbrook, 2001). The customer is then attracted to the brand because of the trust, nostalgia, familiarity, or resistance to change. The favorite brand is prominent in the consumer’s mind in a purchase, and other brands may not be considered relevant (Smith and Brynjolfsson, 2001). The consumer must be persuaded to even consider their properties (Eliaz and Spiegler, 2011). Past consumption thus strongly impacts the current one and preference of a certain brand can persist in the long term (Bronnenberg et al., 2012).

In the same way some kinds of consumption can influence a consumer’s satisfaction even long after the purchase had been made. Memories of it influence the consumer, either positively or negatively, in the following decisions, as they bring an emotional context of the previous purchase into the current decision-making, leading to attribution error. For instance, honeymoon, holiday, or recreation can steer the long-term choices where to go to a new holiday and how to spend it, because the pleasant nostalgia or conversely strong regret will be very prominent in the consumer’s mind. As Simonsohn (2010, p. 272) noted: “[F]oods tasted for the first time on an empty stomach are remembered as more enjoyable and might hence be disproportionately likely to be purchased again.”

Strong primary experiences can produce a similarly strong impact. Children learn consumer behavioral patterns and preferences from their parents, either by direct imitation or shared preferences (Viswanathan et al., 2000). Primary experience can impact car purchases: there is a strong correlation between the brand of cars purchased by parents and their children, even after controlling for shared demographic or geographic characteristics (Anderson et al., 2013). This correlation is strongest for cars purchased at the time when the children had still lived together with their parents. Anderson et al.’s (2013) findings mean that prices of cars for young families can be lower, as the car company creates loyalty in a future generation of customers. Most other studies about inter-generational preference transfer concerns food (Birch, 1999), but research into this phenomenon in other kinds of goods is virtually non-existent.

### Immediate and Frequent Consumption, Anchoring Bias

People are anchored by their immediate or repeated past consumption and/or experience (Simonson and Tversky, 1992). A study (Simonsohn and Loewenstein, 2006) showed that families moving from areas with high rent into cheaper areas spend more on their rent and rent larger homes on average. In contrast, families moving from cheaper into more expensive areas tend to spend less and live in smaller homes (both findings are robust after controlling for the effects of wealth, taste for expensive housing, or mis-estimation of local housing costs etc.). Even after moving, the families were still influenced by their past in their decision about how much they would spend on rent. A similar effect has been found in commuting time; the longer one had commuted in their previous home, the longer they commute in their new location as well (Simonsohn, 2006).

The same anchoring effect, where assessment of a choice is influenced by recent contextually relevant experience, can be found in a number of other situations. Paintings previously sold on “hot” markets for high prices usually continue achieving higher prices in auctions than comparable paintings previously sold in “cold” markets (Beggs and Graddy, 2009). Sellers of houses bought on a growing market, currently facing nominal loss due to unfavorable market situation, keep their houses on the market for a longer time and higher asking price, and eventually sell them for a higher price than sellers who had initially bought comparable houses for lower prices (Genesove and Mayer, 2001). Consumers’ willingness for a purchase can also be affected by completely irrelevant anchors present at the moment of a decision about a purchase (Ariely et al., 2003), however, also see Maniadis et al. (2014) for the criticism of these findings. However, there are not many studies about the dynamics of anchor updating or about particular shopping features, which could (or couldn’t) sway consumers to succumb to an anchor.

### Situational Influences, Emotion, and Mood, Affect Bias

The cognitive frames could be derived from external circumstances and internal psychological states that specifically enhance certain attributes of the product and attract attention to it. As mentioned in the introduction, people prefer insurance against events that come to their mind more easily, not necessarily against events that might have a significant impact on their wellbeing (Gallagher, 2014; Browne et al., 2015).

The local consumer thinking approach can build upon the research of the influence of affect and mood on consumer decision-making (Cohen et al., 2008). People under different emotional states interpret information differently, exhibit different preferences and salient memories, creating specific cognitive frames influencing their behavior (Schwarz, 2000). Consumers’ decision-making is in tow of emotion and visceral factors such as satisfaction, hunger, fear, or exhaustion.

Weather changes are often used as a research tool of the impacts of mood on consumer behavior: during sunny weather, people are happier, while in cloudy conditions, their mood is lower. Restaurants receive worse ratings in rainy weather or heat waves (Bakhshi et al., 2014), sunny weather correlates with higher tips (Rind, 1996) and higher willingness to shop using one’s mobile phone (Reinaker et al., 2015). Results concerning the impact of mood on consumer decision-making show the importance of situational influences on the consumers’ mood (Cohen et al., 2008). There are currently a number of mobile apps tracking or triggering emotional changes, be they weather or social apps (Kramer et al., 2014). They offer new research opportunities for a more detailed identification of the influence of emotional framing in online or mobile shopping.

### Situational Influences and Projection Error

Another example of cognitive frame is that consumers tend to exaggerate the degree to which their future tastes will resemble their current tastes, i.e., they are projecting their current tastes influenced by immediate context to the future, decision-making known as projection bias (Loewenstein et al., 2003). Using catalog orders of cold-weather items Conlin et al. (2007) find that consumers are over-influenced by current weather. If the order-date temperature declines by 30° Fahrenheit, the return probability increases by 3.95%. Weather had moved in a direction that made the item more valuable and/or salient at the time the consumers had been buying, but these biased valuations did not have to hold the same in the future; as a result, the likelihood of returning the item increased. Projection bias affects advance sales for an outdoor movie theater the same way (Buchheim and Kolaska, 2016). Another example is a study by Gorn et al. (1993) demonstrating that in evaluation of a speaker system, customers are influenced by the music currently playing on it.

Projection bias does not emerge only in relatively small value purchases. Days that are warmer and skies that are clearer than seasonal averages influence customers to buy more convertibles. On the contrary, surprising snowstorms increase the sales of cars with four-wheel drive (Busse et al., 2015).

## Conclusion

The theory of consumer local thinking is fairly simple. It states that some information is more salient for the consumers under certain circumstances, drawing disproportional attention to a narrow characteristic of the goods and making even sophisticated and responsible consumers underestimate other, often more relevant information. Future research must specifically identify which attention-drawing measures (i.e., economic, social, and/or environmental sustainability) work, how cost-effective they are and for what kinds of products and customers they work best.

The consumers’ behavioral biases often lead to bad bargains, further exploited by firms to their profit (Grubb, 2015). Despite a body of literature on nudging people toward better decision-making (Thaler and Sunstein, 2009), there are not many real interventions successfully de-biasing consumers in mentioned inept decision-making. Nevertheless, even a small short-term attention shock can improve households’ decision-making. For example, participants of a survey including questions focused on checking account overdraft fees subsequently show a lower probability of checking overdrafts in their real financial decisions (Stango and Zinman, 2014) or see (Bhargava and Manoli, 2015). Future research should examine more techniques to de-biasing some consumers or address the need of regulatory interventions.

## Author CONTRIBUTIONS

The author confirms being the sole contributor of this work and approved it for publication.

## Conflict of Interest Statement

The author declares that the research was conducted in the absence of any commercial or financial relationships that could be construed as a potential conflict of interest.
